# Glucose may Contribute to Retrieval and Reconsolidation of Contextual Fear Memory Through Hippocampal *Nr4a3* and *Bdnf* mRNA Expression and May Act Synergically with Adrenaline

**DOI:** 10.1007/s12035-023-03745-6

**Published:** 2023-11-08

**Authors:** Ana Oliveira, Márcia Azevedo, Rafaela Seixas, Raquel Martinho, Paula Serrão, Mónica Moreira-Rodrigues

**Affiliations:** 1 Department of Immuno-physiology and Pharmacology, Laboratory of General Physiology, School of Medicine and Biomedical Sciences (ICBAS), University of Porto (UP), R. Jorge Viterbo Ferreira, 228, Building 2, Floor 4, Cabinet 22, Porto, 4050-313 Portugal; 2 Center for Drug Discovery and Innovative Medicines, University of Porto (MedInUP), Porto, Portugal; 3 Department of Biomedicine, Faculty of Medicine, University of Porto (FMUP), Porto, Portugal

**Keywords:** Fear conditioning, Contextual fear memory, Glucose, Adrenaline, Adrenaline-deficient mice

## Abstract

Adrenaline (Ad) and glucose released into the bloodstream during stress may strengthen contextual fear memory. However, no previous studies have detached the effects of glucose from Ad in this paradigm. Using Ad-deficient mice, we aimed to evaluate the effect of glucose on contextual fear memory when endogenous Ad is absent. Fear conditioning was performed in wild-type (WT) and Ad-deficient mice (129 × 1/SvJ) administered with glucose (30 or 10 mg/kg; i.p.) or/and Ad (0.01 mg/kg; i.p.) or vehicle (0.9% NaCl; i.p.). Catecholamines were quantified using HPLC-ED. Real-time qPCR was used to assess mRNA expression of hippocampal genes. WT and Ad-deficient mice display increased contextual fear memory when administered with glucose both in acquisition and context days when compared to vehicle. Also, *Nr4a3* and *Bdnf* mRNA expression increased in glucose-administered Ad-deficient mice. Sub-effective doses of glucose plus Ad administered simultaneously to Ad-deficient mice increased contextual fear memory, contrary to independent sub-effective doses. Concluding, glucose may be an important part of the peripheral to central pathway involved in the retrieval and reconsolidation of fear contextual memories independently of Ad, possibly due to increased hippocampal *Nr4a3* and *Bdnf* gene expression. Furthermore, Ad and glucose may act synergically to strengthen contextual fear memory.

## Introduction

Physiological reactions to stress are an essential tool for preserving the homeostasis of the organism: when one is faced with a threat, fear and stress responses enable the organism to react, being responsible for the “fight, flight, or freeze” responses [1]. Humans, as well as other animals, benefit from the ability to retain and use information gained remotely, in order to remember what situations pose a threat to them so they can successfully avoid those situations in the future [2].

However, the processes involved in the acquisition and consolidation of emotional information associated with fear are not always correctly activated. Comprehending how emotional memories, such as fear memories, are formed and stored in the brain allows a window of opportunity for the treatment of pathologies associated with unpleasant and traumatic memories. In fact, while automatic reactions to stress are an important survival tool, when improperly enabled or uncontrolled they can lead to prolonged stress, ultimately leading to a chronic overactivation of the adrenergic system. This overactivation may be implicated in the development of psychiatric disorders such as anxiety, depression, post-traumatic stress disorder (PTSD), and other disorders [3].

Contextual fear conditioning is a behavioural paradigm based on classical conditioning, allowing the association of an aversive stimulus (such as mild-intensity electric shocks) with a specific context. Fear conditioning animal models are frequently used in research to study the neurobiological basis of adverse memories, being able to indicate the presence of fear and anxiety. These behaviour variations possibly reflect alterations in certain brain regions such as the hippocampus and the amygdala, which interplay together for contextual fear memory formation [4]. For instance, memory acquisition of the context occurs in the hippocampus and the memory acquisition of the emotional information (fear in the case of fear conditioning) takes place in the amygdala [5–9].

Contextual fear conditioning provokes stress and activates the hypothalamus-pituitary-adrenal axis, which triggers the release of catecholamines, in particular noradrenaline (NA) and adrenaline (Ad) from the adrenal gland into the bloodstream [10]. Moreover, several authors have shown that catecholamines are important for long-term memory consolidation in human and animal studies, in which Ad administration immediately after training sessions increased memory retention compared to vehicle administration [11, 12]. Furthermore, in previous studies, we and others have demonstrated that mice not capable of expressing the enzyme phenylethanolamine-*N*-methyltransferase (Pnmt), which catalyses the conversion of NA to Ad, and, therefore, are adrenaline-deficient (Ad-deficient, Pnmt-KO) [13], have reduced contextual fear memory [14, 15]. In addition, Ad strengthens contextual fear memory and induces, in the hippocampus, the expression of genes related to contextual fear memory consolidation, such as transcription factor nuclear receptor 4a2 (*Nr4a2*) [10].

Despite all the evidence for Ad enhancement of contextual fear memory, as a hydrophilic catecholamine, peripheral Ad does not cross the blood-brain barrier (BBB) which prevents it from acting in the central nervous system (CNS) [16]. Therefore, the role of Ad in contextual fear memory appears to be due to its peripheral actions rather than direct central ones. This suggestion led several authors to propose that this catecholamine may exert its central actions through glucose. Consistently with this theory, Morris and Gold proposed the hypothesis that glucose may mediate Ad action in the CNS [17].

Since Ad induces blood glucose increase, it is difficult to evaluate the effects of glucose detached from those of Ad in contextual fear memory. Thus, using Ad-deficient mice (do not synthesize Ad), which is a reliable model to better understand Ad and glucose effects alone on contextual fear memory, the main aim of this study was to detach the effects of Ad from those of glucose and evaluate their action on contextual fear memory strengthening through possible effects in the CNS. Therefore, we attempted to evaluate if glucose administration enhances contextual fear memory in an Ad-deficient mouse model, as well as to evaluate a possible synergy between Ad and glucose. Lastly, another aim of this study was to evaluate the hippocampal gene expression implications of glucose administration in Ad-deficient mice after fear conditioning.

## Methods

### Animals

The animal care and experimental protocols were performed in accordance with Portuguese legislation by Directive Law 113/2013 and 1/2019 and approved by the Organism Responsible for Animal Welfare (ORBEA) in the Faculty of Medicine of the University of Porto and National Authority for Animal Health (DGAV) which is a transposition of the European Directive number 2010/63/EU. In the vivarium, Ad-deficient (Pnmt-KO) (*n* = 49) and WT (*n* = 27) male mice (129 × 1/SvJ) were bred under controlled environmental conditions (12 h light/dark cycle, room temperature 23 ± 1 °C, 50% of humidity, *ad libitum* autoclaved drinking water and mice diet 4RF25/I and 4RF21/A; Mucedola, Milan, Italy) in the same room and housed with the respective litter, in cages with two to five animals.

A Cre-recombinase gene and a Neomycin resistance gene were inserted into exon 1 of the locus encoding for *Pnmt* gene, resulting in the Ad-deficient (*Pnmt*^*−/−*^, Pnmt-KO) mice. These *Pnmt*^*−/−*^ mice are unable to express the Pnmt enzyme and, therefore, unable to produce Ad [13]. This was confirmed by polymerase chain reaction (PCR) of DNA ear samples, as previously shown [13].

### Blood Glucose Quantification

**Fig. 1:**
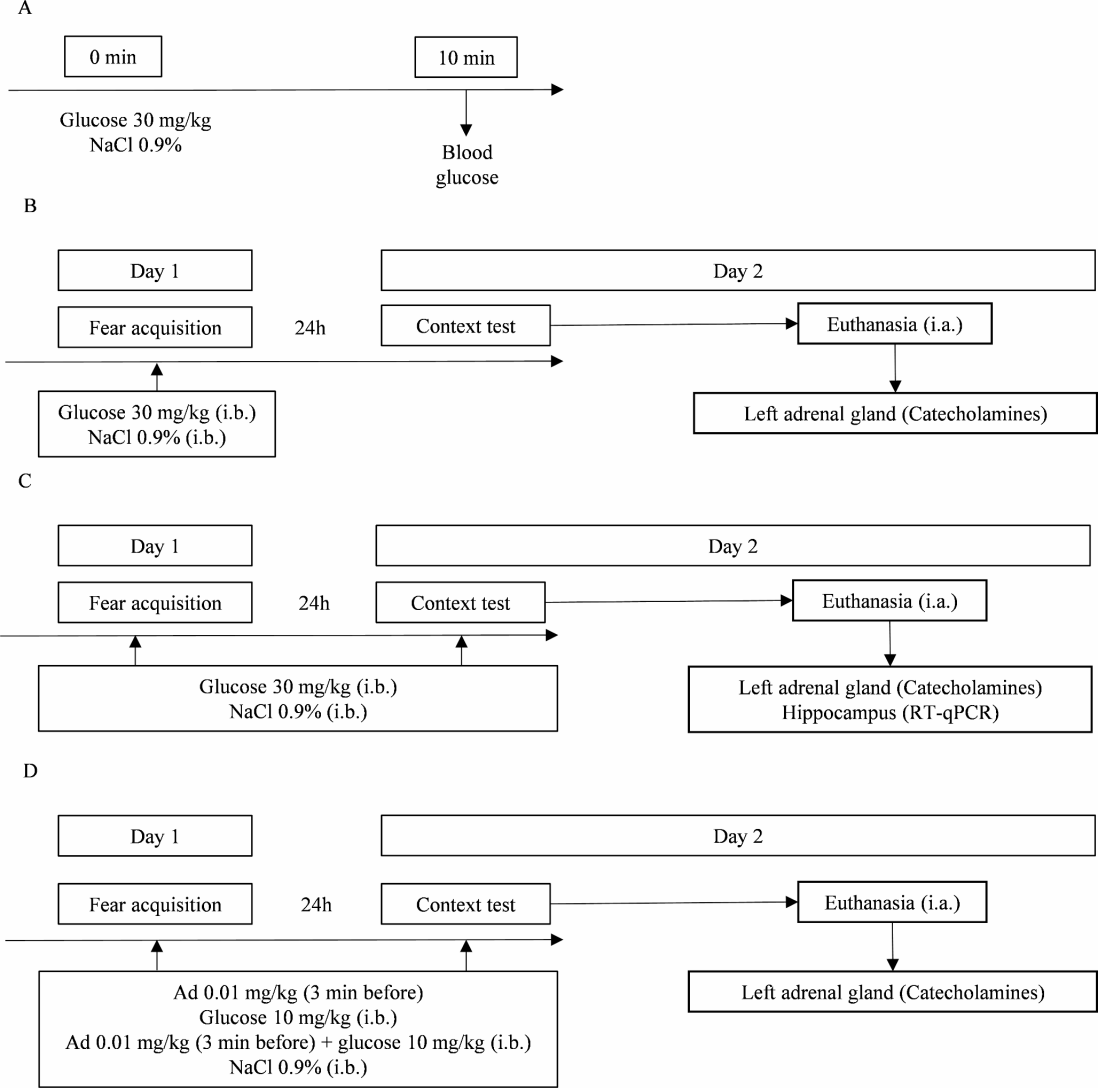
Schematic representation of the experimental design, behavioural protocols, treatments, and sample collection. **(A)** Initially, a group of wild-type (WT) mice followed protocol A being administered with glucose (30 mg/kg; intraperitoneal, i.p.) or vehicle (0.9% NaCl; i.p.) and 10 min after blood glucose was measured. Afterwards, mice were submitted to fear conditioning protocol with different experimental protocols: **(B)** A group of WT mice followed protocol B being administered with glucose (30 mg/kg; i.p., immediately before, i.b.) or vehicle (0.9% NaCl; i.p., i.b.) only on the fear acquisition day. **(C)** Subsequently, a group of WT and Ad-deficient (Pnmt-KO) mice followed protocol C with glucose (30 mg/kg; i.p.; i.b.) or vehicle (0.9% NaCl; i.p., i.b.) administration in both the fear acquisition and context days. **(D)** Finally, a group of Ad-deficient (Pnmt-KO) mice followed protocol D administered in both the fear acquisition and context days with sub-effective doses of Ad (0.01 mg/kg; i.p., 3 min), glucose (10 mg/kg; i.p., i.b.), Ad (0.01 mg/kg; i.p., 3 min) plus glucose (10 mg/kg; i.p., i.b.), or vehicle (0.9% NaCl; i.p., i.b.). RT-qPCR, real-time polymerase chain reaction. i.a, immediately after

Initially, a group of WT mice was administered with glucose (30 mg/kg; intraperitoneally, i.p.) or vehicle (NaCl 0.9%, i.p.). Ten minutes after administration and following local anaesthesia (Lidonostrum®) applied on the site of the needle prick, a blood glucose drop was collected in a blood glucose test strip (FreeStyle®) connected to a glucose meter (FreeStyle®) for blood glucose concentration (mg/dl) quantification (Fig. 1A) [18].

### Conditioning Fear Test

The procedure followed for the conditioned fear test was adapted from previous studies [10, 14]. The conditioning chamber consisted of a clear Plexiglas box (26.5 cm x 26.5 cm x 26.5 cm) equipped with a metal grid floor with 32 stainless steel bars (placed 5 mm apart) wired to a stimulus generator. The first day of the conditioned fear test (fear acquisition) had a duration of 6 min: the first 3 min consisted of a habituation period (mice left undisturbed), after which 3-foot shocks (duration: 2 s, intensity: 0.6 mA) were delivered at 60 s intervals. After 24 h, mice were re-exposed to the same apparatus with no foot shocks delivered during the total duration of the context test (10 min; context). The 10-minute duration of the context was chosen to allow hippocampal gene expression after re-exposure [19]. A timer was positioned on top of the contextual chamber and both days were video recorded with a digital video camera Sony HDR-CX405 (Sony Corporation, Japan) to allow the posterior evaluation of the mice’s behaviour. All analyses were conducted under manual and blind protocols. On acquisition and context days, freezing behaviour, defined as the lack of movements excluding those related to respiration for at least 3 s, was evaluated [20]. Additionally, on the acquisition day, vocalization (high-pitched squeak) and jump (removal of 3 paws from the grid floor) responses to the foot shock were also quantified [21]. On both days, the conditioned chamber was cleaned with alcohol 70% and smeared with 1% acetic acid between trials.

### Behavioural Treatments

WT and Ad-deficient (Pnmt-KO) mice were submitted to the fear conditioning protocol with different experimental designs as displayed in Fig. 1. A group of WT mice were administered pre-acquisition with glucose (30 mg/kg, i.p.; immediately before, i.b.) or vehicle (0.9% NaCl, i.p., i.b.) (Fig. 1B). Next, a group of WT or Ad-deficient (Pnmt-KO) mice were administered pre-training and pre-context with glucose (30 mg/kg, i.p., i.b.) or vehicle (0.9% NaCl, i.p., i.b.) (Fig. 1C). Finally, a group of Ad-deficient (Pnmt-KO) mice were administered with Ad (0.01 mg/kg, i.p., 3 min), glucose (10 mg/kg, i.p., i.b.), Ad (0.01 mg/kg, i.p., 3 min) plus glucose (10 mg/kg, i.p., i.b.), or vehicle (0.9% NaCl, i.p., i.b.) (Fig. 1D).

### Quantification of Catecholamines

Mice were anaesthetized (ketamine, 100 mg/kg and xylazine, 10 mg/kg; i.p.) immediately after the end of the fear conditioning procedure for samples and tissues collection. The left adrenal gland was collected and emerged in perchloric acid (PCA) 0.2 M overnight, at 4 ºC, and frozen at -80 ºC until further use. The PCA of each sample was transferred to tubes with filters and centrifuged at 1250 g for 2 min at 4 ºC to quantify catecholamines in the adrenal gland. An external standard solution (125 mg/µl for purified Ad, NA, and DA (Sigma-Aldrich, St. Louis, USA) was used. Afterwards, 50 µL of the PCA of each sample or standard solution were mixed with the mobile phase (citric acid monohydrate 100 mM, sodium acetate 100 mM, 1-octanesulfonic acid sodium salt 1.6 mM, ethylenediaminetetraacetic acid (EDTA) disodium salt dihydrate 0.15 mM, methanol 8%, di-n-butylamine 1 mM, pH was set for 3.5 with HClO_4_ 70%) and injected in a reverse-phase high-performance liquid chromatography (HPLC) (Gilson Medical Electronics, Villiers le Bel, France) machine. The components of interest in each sample or in the external standard solution were separated in the column (SPHERI-5 C18 4.6 × 250 mm, 5 μm; BROWNLEE™, Waltham, MA, USA), and quantified by electrochemical detection (ED). The limits of detection and quantification for the HPLC (Gilson Medical Electronics, Villiers le Bel, France) employed for adrenal gland samples were 350–1000 fmol and 700–2000 fmol, respectively. NA = 6.60 min, Ad = 7.81 min, and DA = 15.95 min were the retention times. Catecholamine content was calculated per whole adrenal gland (nmol/AG).

### RNA Isolation and Relative Quantification of mRNA Expression

Real-time PCR (qPCR) was performed in hippocampus samples collected immediately after the context day of the fear conditioning protocol, as described in previous works [10, 22, 23]. Total RNA isolation was carried out with Mini Kit illustra™ Isolate II RNA (Bioline, London, United Kingdom), according to the manufacturer’s instructions. In, brief, ethanol (70%) was added to the tubes containing the RNA. Afterwards, desalting was performed by adding a Membrane Desalting Buffer (MEM) (Bioline, London, United Kingdom) to the tubes. For DNA digestion, DNase I (Bioline, London, United Kingdom) was added to the Reaction Buffer for DNase I (RDN) (Bioline, London, United Kingdom). Next, the DNase I reaction mixture was applied directly to the center of the silica membrane. Three washes were then performed. At first, the RNA was washed with Wash Buffer RW1 (Bioline, London, United Kingdom). For the second and third washes it was used Wash Buffer RW2 (Bioline, London, United Kingdom). Finally, RNA was eluted with RNase-free water (Bioline, London, United Kingdom). The concentration and purity of the isolated RNA were measured with NanoDrop 2000 spectrophotometer (Thermo Scientific, Waltham, United States). A T100™ Thermal Cycler (Bio-Rad, Hercules, United States) was used to carry out reverse transcription, applying a Reverse Transcription kit (NZY First-Strand cDNA Synthesis Kit NZYTech — Genes and Enzymes, Lisbon, Portugal). StepOne™ real-time PCR System (Applied BioSystems, Waltham, United States) allowed the conduction of qPCR reactions. Gene-specific primers (10 µM), Maxima SYBR Green qPCR Master Mix (Thermo Scientific, Waltham, MA, United States), RNase-free H_2_O (Bioline, London, United Kingdom) were mixed and cDNA was added (1:20). Instead of cDNA, RNase-free H_2_O (Bioline, London, United Kingdom) was added as a negative control. Gene-specific primers are presented in Table 1. Results of mRNA quantification were expressed in an arbitrary unit (AU) after normalization for Glyceraldehyde 3-phosphate dehydrogenase (GAPDH).

**Table 1:** Primers used in gene expression analysis

Gene	Primer (5’→ 3’)
*Nr4a1*	F: AAAATCCCTGGCTTCATTGAG
	R: TTTAGATCGGTATGCCAGGCG
*Nr4a2*	F: CGGTTTCAGAAGTGCCTAGC
	R: TTGCCTGGAACCTGGAATAG
*Nr4a3*	F: GTGGCTCGACTCCATTAAAGAC
	R: GTGCATAGCTCCTCCACTCTCT
*Bdnf*	F: GGACATATCCATGACCAGAAAGAAA
	R: GCAACAAACCACAACATTATCGAG
*Gapdh*	F: CCATCACCATCTTCCAGGAG
	R: GCATGGACTGTGGTCATGAG

Nr4, Nuclear receptor 4; Bdnf, Brain-derived neurotrophic factor; Gapdh, Glyceraldehyde 3-phosphate dehydrogenase; F, forward primer and R, reverse primer

### Drugs

D (+)-Glucose was purchased from EMD Millipore Corporation (Germany); (-) - adrenaline, L-(-)-noradrenaline, dopamine hydrochloride, and PCA were purchased from Sigma-Aldrich (St. Louis, United States). Ketamine (Imalgene 1,000, Merial, Lisbon, Portugal) and xylazine (Rompum 2%, Bayer, Lisbon, Portugal) were purchased from Agrofauna (Vila Nova de Gaia, Portugal). Lidocaine hydrochloride (Lidonostrum® 2%, Sidefarma, Lisbon, Portugal).

### Statistical Analysis

An online Sample Size Calculator was used to establish the minimum sample size for each experimental protocol (https://clincalc.com/Stats/SampleSize.aspx). All statistical analysis was performed using the program GraphPad Prism (GraphPad Software Inc., La Jolla, CA, USA), and all data was displayed as means with standard error of the means (SEM). Freezing behaviour was analysed by Two-Way Analysis of Variance (ANOVA) repeated measures, followed by multiple comparison corrections with Sidak’s (two groups) or Tukey´s (three or more groups) post-hoc tests using treatment as a “between-subjects factor” and time as “within-subjects factor” (repeated measure) (α was set at 0.05). Other behavioural outcomes (vocalization and jump responses), catecholamines concentration, and qPCR were examined using Student’s t-test (2 groups) or One-Way ANOVA (three or more groups). For One-Way ANOVA the post-hoc Tukey test was used for multiple comparisons correction (α was set at 0.05). Effect sizes were estimated by calculating Cohen’s *d* test for t-test comparisons and partial eta squared (*η*_*2*_^*p*^) for ANOVA. The statistically significant differences observed between groups in the different time points or between groups in Two-way and One-way ANOVA, respectively, were based on the adjusted *p-*value < 0.05. The statistically significant difference for unpaired t-test analysis was set at *p* < 0.05.

## Results

### Hyperglycaemic State is Attained 10 Min After Glucose Administration

**Fig. 2:**
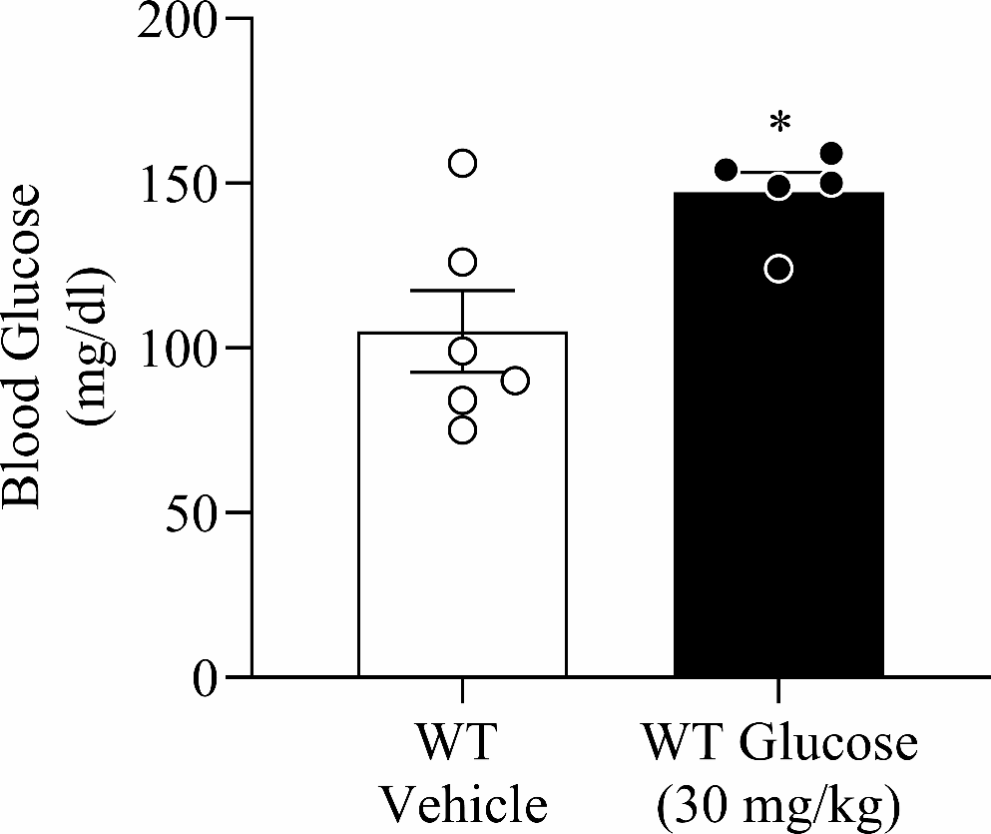
Blood glucose concentration in wild-type mice ten minutes after glucose administration. Wild-type (WT) mice were administered with glucose (30 mg/kg; intraperitoneal, i.p.) or vehicle (NaCl 0.9%; i.p.). Values are means ± SEM of 5–6 mice per group. WT Glucose, wild-type mice administered with glucose; WT Vehicle, wild-type mice administered with vehicle. *, significantly different from correspondent values in WT Vehicle mice (*p* < 0.05)

In an initial approach (Fig. 1A), it was verified a significant increase in blood glucose when compared with vehicle-treated mice (t _(9)_ = 2.85, *p =* 0.02, Cohens’ *d* = 1.97, Fig. 2). Therefore, it was confirmed that glucose-administered WT mice attained a moderate hyperglycaemic state 10 min after glucose (30 mg/kg) administration. This preliminary evaluation gave us information for all the subsequent protocols which were carried out considering the glucose concentration and time point identified.

### Glucose Administration Only Before the Fear Acquisition Day Does not Strengthen Contextual Fear Memory in Wild-Type Mice

**Fig. 3:**
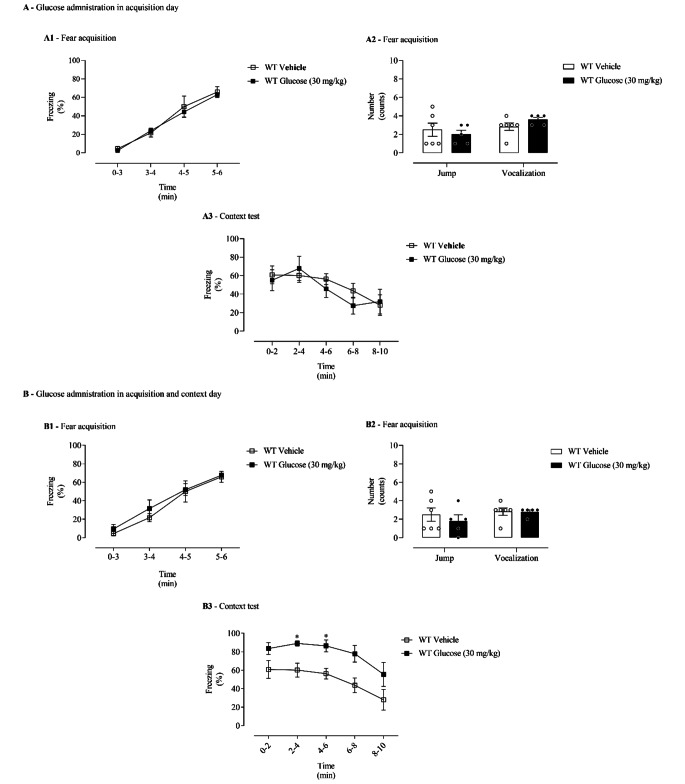
Fear behaviour in wild-type mice after glucose administration. Glucose (30 mg/kg; intraperitoneal, i.p.) or vehicle (NaCl, 0.9%; i.p.) administration **(A)** only on the fear acquisition day and **(B)** in both the fear acquisition and context days. (A1 and B1) Freezing and (A2 and B2) shock responsivity in fear acquisition day. (A3 and B3) Freezing response in re-exposure to the context. Values are means ± SEM of 5–6 mice per group. WT Glucose, wild-type mice administered with glucose; WT Vehicle, wild-type mice administered with vehicle. *, significantly different from correspondent values in WT Vehicle mice (adjusted *p*-value < 0.05)

Figure 3A shows the effects in freezing behaviour and shock responsiveness of glucose (30 mg/kg) administration only on the fear acquisition day, in WT mice (Fig. 1B). In acquisition day, it was observed a time effect (F _(3, 27)_ = 58.39, *p* = 6.26 × 10^− 12^, ƞ_p_^2^ = 0.87, Fig. 3A1), but no significant effect of treatment (F _(1, 9)_ = 0.13, *p =* 0.72, ƞ_p_^2^ = 0.01, Fig. 3A1) or time x treatment interaction (F _(3, 27)_ = 0.22, *p =* 0.88, ƞ_p_^2^ = 0.02, Fig. 3A1) were present. No differences between glucose or vehicle-administered WT mice were observed in the freezing response (Fig. 3A1). Also, no differences were observed in vocalization (t _(9)_ = 1.55, *p =* 0.16, Cohens’ *d* = 1.06, Fig. 3A2) and jump (t _(9)_ = 0.56, *p =* 0.59, Cohens’ *d* = 0.38, Fig. 3A2) responses on the first day of the fear conditioning protocol (training day).

On context day, after re-exposure to shock context, a significant effect of time (F _(4, 36)_ = 16.69, *p* = 8.02 × 10^− 8^, ƞ_p_^2^ = 0.65, Fig. 3A3) was observed, but no effect of treatment (F _(1, 9)_ = 0.11, *p* = 0.74, ƞ_p_^2^ = 0.01, Fig. 3A3) or time x treatment interaction (F _(4, 36)_ = 1.95, *p =* 0.12, ƞ_p_^2^ = 0.18, Fig. 3A3) were present. Administration with glucose on acquisition day does not alter freezing behaviour in WT mice compared with vehicle-administered WT mice (Fig. 3A3).

No differences were observed in dopamine (DA) (t _(9)_ = 1.80, *p =* 0.10, Cohens’ *d* = 1.22, Table 2) and NA (t _(9)_ = 1.86, *p =* 0.09, Cohens’ *d* = 1.28, Table 2) content in the adrenal gland between groups. Ad increased in glucose-administered WT mice compared to vehicle (t _(9)_ = 2.53, *p =* 0.03, Cohens’ *d* = 1.73, Table 2).

**Table 2:** Adrenal gland catecholamines’ concentration in wild-type (WT) mice administered with glucose before (A) only the acquisition day and (B) both the acquisition and context days

Administration days	Treatment groups	DA (nmol/AG)	NA (nmol/AG)	Ad (nmol/AG)
(A) Acquisition	WT Vehicle (NaCl 0.9%)	0.29 ± 0.04	6.39 ± 0.76	14.27 ± 1.62
	WT Glucose (30 mg/kg)	0.42 ± 0.06	8.11 ± 0.43	19.45 ± 1.11*
(B) Acquisition and context	WT Vehicle (NaCl 0.9%)	0.28 ± 0.05	6.18 ± 0.90	13.77 ± 1.88
	WT Glucose (30 mg/kg)	0.30 ± 0.03	6.08 ± 0.35	17.56 ± 0.86

Values are means ± standard error of the means (SEM) of 5–6 mice per group. WT Glucose, wild-type mice administered with glucose; WT Vehicle, wild-type mice administered with vehicle; DA, dopamine; NA, noradrenaline; Ad, adrenaline. *, significantly different from correspondent values in WT Vehicle mice (*p* < 0.05)

### Glucose Administration Both Before the Fear Acquisition and Context Days Appears to Strengthen Contextual Fear Memory in Wild-Type Mice

Figure 3B shows the effects in freezing behaviour and shock responsiveness of glucose (30 mg/kg) administrated both in the fear acquisition and context days (Fig. 1C), in WT mice. In acquisition day, it was observed a time effect (F _(3, 27)_ = 40.82, *p* = 3.60 × 10^− 10^, ƞ_p_^2^ = 0.82, Fig. 3B1), but no significant effect of treatment (F _(1, 9)_ = 0.57, *p =* 0.47,, ƞ_p_^2^ = 0.06, Fig. 3B1) or time x treatment interaction (F _(3, 27)_ = 0.21, *p =* 0.89, ƞ_p_^2^ = 0.02, Fig. 3B1) were present. No differences between the groups were observed in the freezing response (Fig. 3B1). Also, no differences were observed in vocalization (t _(9)_ = 0.07, *p =* 0.95, Cohens’ *d* = 0.05, Fig. 3B2) and jump (t _(9)_ = 0.70, *p =* 0.50, Cohens’ *d* = 0.48, Fig. 3B2) responses on the first day of the fear conditioning protocol.

On context day, during re-exposure to shock context, a significant effect of time (F _(4, 36)_ = 14.20, *p* = 4.76 × 10^− 7^, ƞ_p_^2^ = 0.61, Fig. 3B3) and treatment (F _(1, 9)_ = 7.84, *p* = 0.02, ƞ_p_^2^ = 0.47, Fig. 3B3) was observed. Glucose (30 mg/kg) administration increased freezing behaviour in WT mice compared with vehicle-treated WT mice (Fig. 3B3).

No differences were observed in DA (t _(9)_ = 0.30, *p =* 0.77, Cohens’ *d* = 0.21, Table 2), NA (t _(9)_ = 0.12, *p =* 0.91, Cohens’ *d* = 0.08, Table 2), and Ad (t _(9)_ = 1.94, *p =* 0.08, Cohens’ *d* = 1.28, Table 2) content in the adrenal gland when comparing the experimental groups.

### Glucose Administration Appears to Strengthen Contextual Fear Memory in Adrenaline-Deficient Mice

**Fig. 4:**
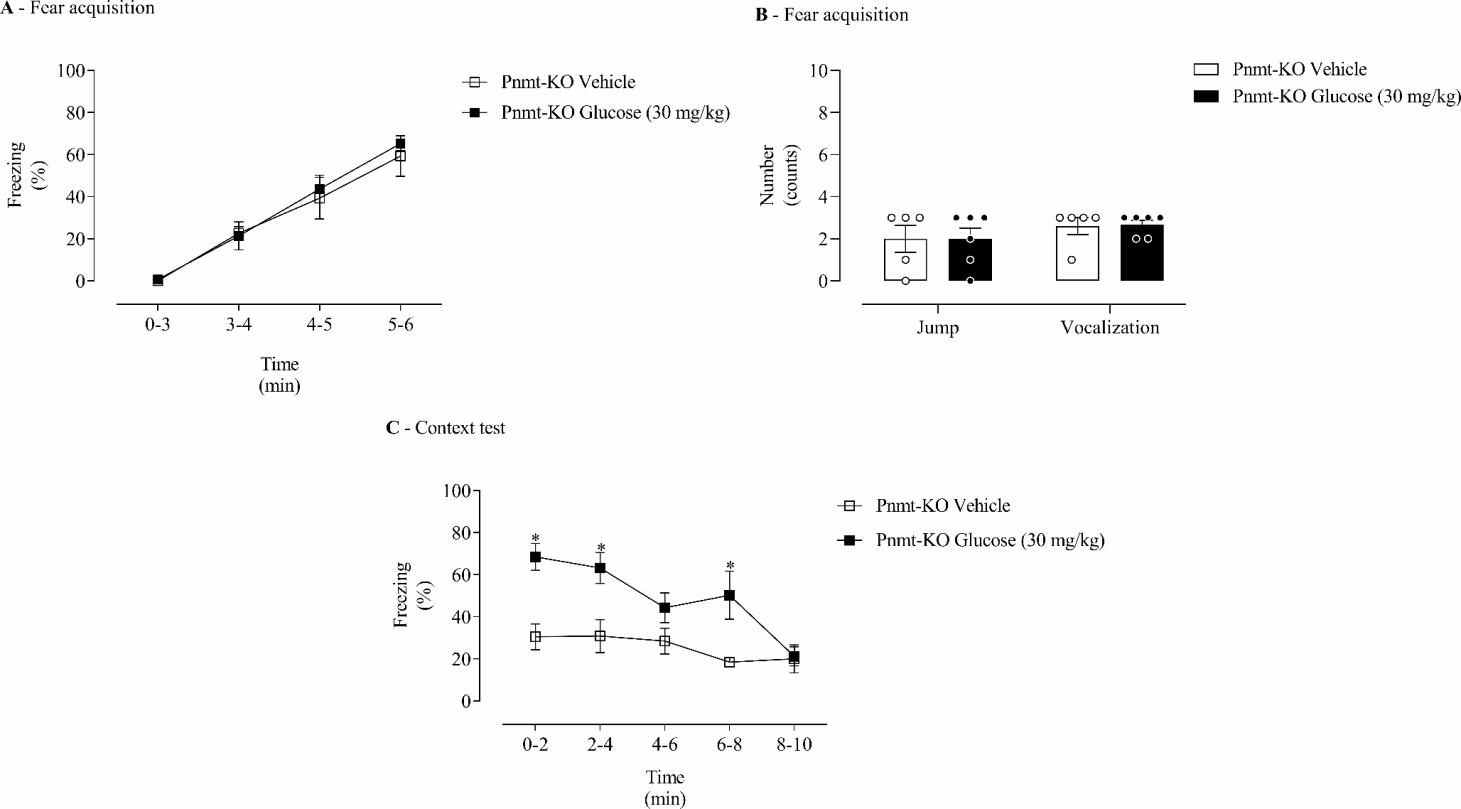
Fear behaviour in adrenaline-deficient mice after glucose administration in both the fear acquisition and context days. **(A)** Freezing and **(B)** shock responsivity on fear acquisition day, and **(C)** freezing response in re-exposure to the context in Ad-deficient (Pnmt-KO) mice administered pre-training and pre-testing with glucose (30 mg/kg; intraperitoneal, i.p.) or vehicle (NaCl, 0.9%; i.p.). Values are means ± SEM of 5–6 mice per group. Pnmt-KO Glucose, adrenaline-deficient mice treated with glucose; Pnmt-KO Vehicle, adrenaline-deficient mice treated with vehicle. *, significantly different from correspondent values in Pnmt-KO Vehicle mice (adjusted *p*-value < 0.05)

To understand the individual effect of glucose independently from Ad, Ad-deficient (Pnmt-KO) mice were administered pre-acquisition and pre-context with glucose (30 mg/kg) and submitted to contextual fear conditioning protocol (Fig. 1C). On acquisition day, a time effect (F _(3, 27)_ = 59.56, *p* = 4.97 × 10^− 12^, ƞ_p_^2^ = 0.87, Fig. 4A) was observed. No significant effect of treatment (F _(1, 9)_ = 0.16, *p* = 0.70, ƞ_p_^2^ = 0.02, Fig. 4A) and time x treatment interaction (F _(3, 27)_ = 0.23, *p* = 0.87, ƞ_p_^2^ = 0.02, Fig. 4A) was observed. No differences were observed in freezing response between treatments on the fear acquisition day (Fig. 4A). Also on the fear acquisition day, vocalization (t _(9)_ = 0.16, *p =* 0.88, Cohens’ *d* = 0.10, Fig. 4B) and jump (t _(9)_ = 0.00, *p =* 1.00, Cohens’ *d* = 0.00, Fig. 4B) responses were not different between treatment groups.

Once re-exposed to shock context, a treatment (F _(1, 9)_ = 13.40, *p* = 0.005, ƞ_p_^2^ = 0.60, Fig. 4C) and time (F _(4, 36)_ = 7.08, *p* = 0.0003, ƞ_p_^2^ = 0.44, Fig. 4C) effect, and a significant time x treatment interaction between treatment and time (F _(4, 36)_ = 3.04, *p* = 0.003, ƞ_p_^2^ = 0.25, Fig. 4C) was observed. Ad-deficient (Pnmt-KO) mice administered with glucose presented an increase in freezing behaviour compared to vehicle-treated Ad-deficient (Pnmt-KO) mice (Fig. 4C).

Across groups, there were no differences in DA (t _(9)_ = 0.76, *p =* 0.11, Cohens’ *d* = 1.18, Table 3) and NA content (t _(9)_ = 0.71, *p =* 0.50, Cohens’ *d* = 0.48, Table 3) in the adrenal gland. In Ad-deficient mice (Pnmt-KO) no Ad was detected by HPLC-ED (Table 3).

**Table 3:** Adrenal gland catecholamines’ concentration in adrenaline-deficient (Pnmt-KO) mice administered with glucose before both the acquisition and context days

Treatment groups	DA (nmol/AG)	NA (nmol/AG)	Ad (nmol/AG)
Pnmt-KO Vehicle (NaCl 0.9%)	0.23 ± 0.02	16.20 ± 1.14	Undetectable
Pnmt-KO Glucose (30 mg/kg)	0.20 ± 0.02	14.41 ± 1.07	Undetectable

Values are means ± standard error of the means (SEM) of 5–6 mice per group. Pnmt-KO Glucose, adrenaline-deficient mice administered with glucose; Pnmt-KO vehicle, adrenaline-deficient mice administered with vehicle; DA, dopamine; NA, noradrenaline; Ad, adrenaline

### Glucose Administration Increases *Nr4a3* and *Bdnf *mRNA Hippocampal Gene Expression in Adrenaline-Deficient Mice

**Fig. 5:**
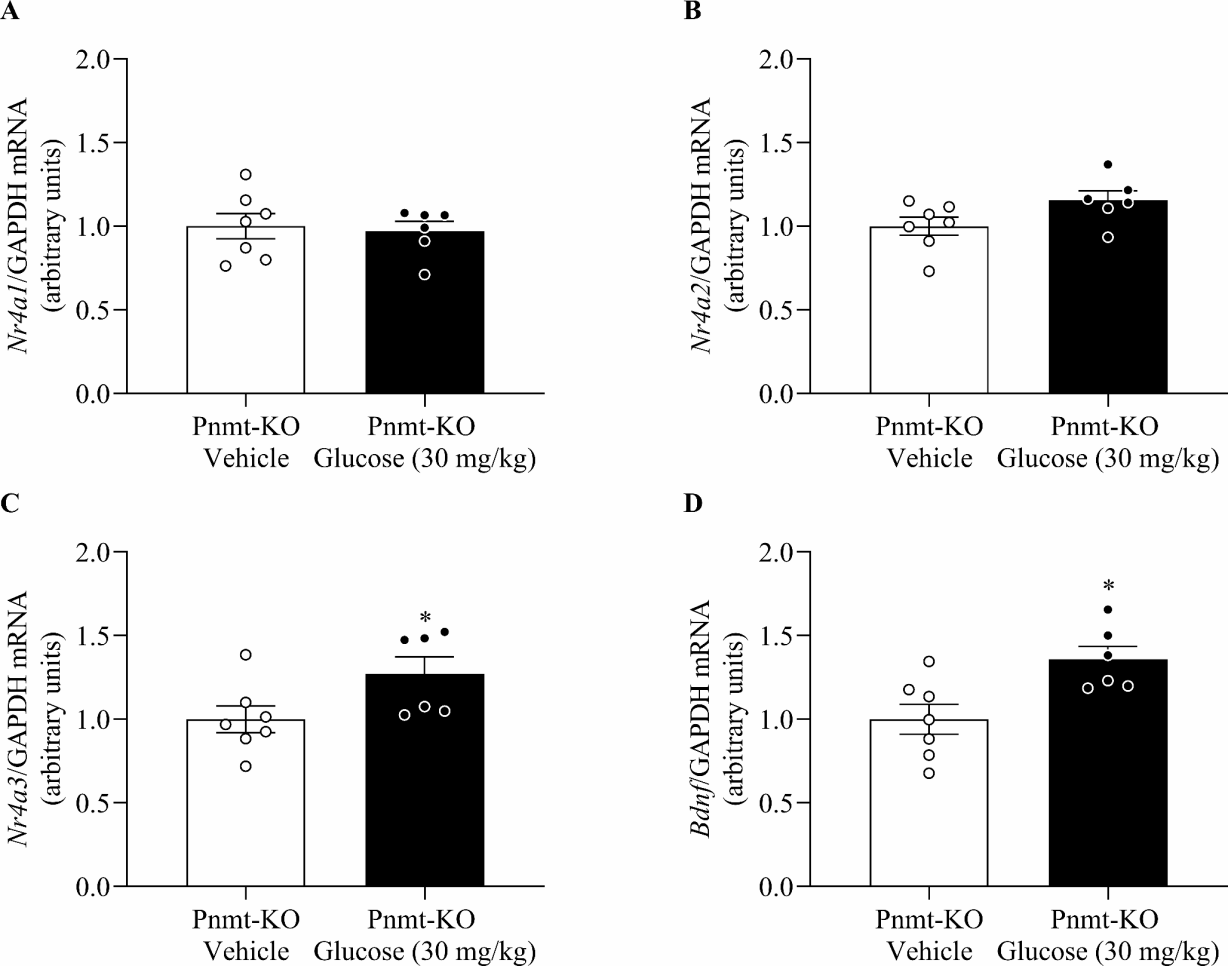
Hippocampus mRNA expression of transcription factor genes after glucose administration in adrenaline-deficient mice. Hippocampus mRNA expression of **(A)** Nuclear receptor 4 (Nr4) a1, **(B)***Nr4a2*, **(C)***Nr4a3*, and **(D)***Bdnf* in adrenaline-deficient (Pnmt-KO) mice after glucose (30 mg/kg; i.p.) or vehicle (NaCl 0.9%; i.p.) administration in both the fear acquisition and context days. Values are means ± SEM of 6–7 mice per group. Results of mRNA are expressed as arbitrary units (AUs) after normalization for glyceraldehyde 3-phosphate dehydrogenase (GAPDH). Pnmt-KO Glucose, adrenaline-deficient mice treated with glucose; Pnmt-KO Vehicle, adrenaline-deficient mice treated with vehicle. *, significantly different from correspondent values in Pnmt-KO vehicle mice (*p* < 0.05)

The influence of glucose in the hippocampal gene expression independently from Ad was evaluated (Fig. 1C). No differences were observed in hippocampus mRNA expression of *Nr4a1* (t _(11)_ = 0.29, *p =* 0.77, Cohens’ *d* = 0.18, Fig. 5A) and *Nr4a2* (t _(11)_ = 1.96, *p =* 0.08, Cohens’ *d* = 1.18, Fig. 5B) between groups. However, the administration of Glucose (30 mg/kg) in Ad-deficient (Pnmt-KO) mice increased hippocampus mRNA expression of *Nr4a3* (t _(11)_ = 2.18, *p =* 0.05, Cohens’ *d* = 1.31, Fig. 5C) and *Bdnf* (t _(11)_ = 2.99, *p =* 0.01, Cohens’ *d* = 1.82, Fig. 5D) compared with vehicle-treated Ad-deficient (Pnmt-KO) mice.

### Glucose and Adrenaline Appear to Act Synergically to Strengthen Contextual Fear Memory in Adrenaline-Deficient Mice

**Fig. 6:**
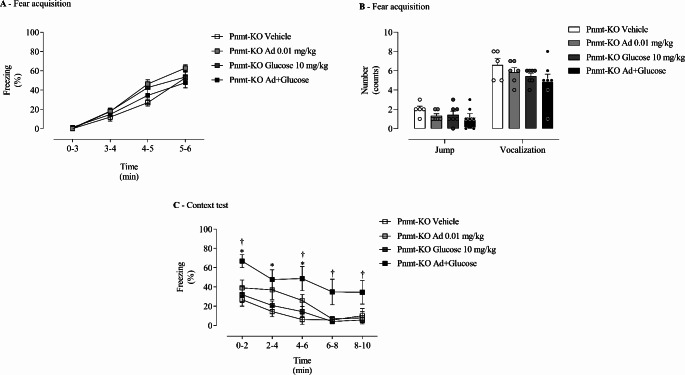
Fear behaviour in adrenaline-deficient mice after glucose or/and adrenaline sub-effective dose administration. **(A)** Freezing and **(B)** shock responsivity on fear acquisition day, and **(C)** freezing response in re-exposure to the context in Ad-deficient (Pnmt-KO) mice administered pre-training and pre-testing with glucose (10 mg/kg; intraperitoneal, i.p.), Ad (0.01 mg/kg; i.p.), glucose (10 mg/kg; i.p.) plus Ad (0.01 mg/kg; i.p.), or vehicle (NaCl, 0.9%; i.p.). Values are means ± SEM of 5–7 mice per group. Pnmt-KO Glucose, adrenaline-deficient mice treated with glucose; Pnmt-KO Ad, adrenaline-deficient mice treated with adrenaline; Pnmt-KO Glucose + Ad, adrenaline-deficient mice treated with glucose plus adrenaline; Pnmt-KO Vehicle, adrenaline-deficient mice treated with vehicle. *, significantly different from correspondent values in Pnmt-KO vehicle mice (adjusted *p*-value < 0.05). †, significantly different from correspondent values in Pnmt-KO Ad mice (adjusted *p*-value < 0.05)

To infer an interaction between glucose and Ad in contextual fear memory, Ad-deficient (Pnmt-KO) mice were submitted to fear conditioning protocol after treatment with sub-effective doses (Fig. 1D). On the fear acquisition day, it was observed a time effect (F _(3, 63)_ = 156.5, *p =* 6.81 × 10^− 36^, ƞ_p_^2^ = 0.88, Fig. 6A). No significant effect of treatment (F _(3, 21)_ = 1.11, *p* = 0.37, ƞ_p_^2^ = 0.14, Fig. 6A) and time x treatment interaction (F _(9, 63)_ = 1.33, *p* = 0.24, ƞ_p_^2^ = 0.03, Fig. 6A) was observed. No differences were observed in the freezing response between groups (Fig. 6A). Furthermore, no differences were observed in vocalization (F _(3, 21)_ = 0.62, *p =* 0.61, ƞ_p_^2^ = 0.08, Fig. 6B) and jump (F _(3, 21)_ = 0.57, *p =* 0.63, ƞ_p_^2^ = 0.08, Fig. 6B) responses between treatments.

After re-exposure to the shock context, a treatment (F _(3, 21)_ = 5.96, *p* = 0.004, ƞ_p_^2^ = 0.46, Fig. 6C) and time (F _(4, 84)_ = 18.35, *p* = 7.23 × 10^− 11^, ƞ_p_^2^ = 0.47, Fig. 6C) effect were observed. No differences were observed between Ad-deficient (Pnmt-KO) mice treated with a sub-effective dose of Ad (0.01 mg/kg) or Glucose (10 mg/kg) and vehicle (Fig. 6C). However, when Ad-deficient (Pnmt-KO) mice were treated simultaneously with Ad (0.01 mg/kg) and Glucose (10 mg/kg) it was observed an increase in freezing behaviour when compared to Ad-deficient (Pnmt-KO) mice only treated with Ad (0.01 mg/kg), or Glucose (10 mg/kg) or vehicle (Fig. 6C).

Concerning the adrenal glands, no differences were observed in DA (F _(3, 21)_ = 1.27, *p =* 0.31, ƞ_p_^2^ = 0.15, Table 4) or NA content (F _(3, 21)_ = 0.43, *p =* 0.74, ƞ_p_^2^ =0.06, Table 4) between groups. In Ad-deficient mice (Pnmt-KO) no Ad was detected by HPLC-ED (Table 3).

**Table 4:** Adrenal gland catecholamines’ concentration in adrenaline-deficient (Pnmt-KO) mice administered with sub-effective doses of glucose and/or adrenaline before both the acquisition and context days

Treatment groups	DA (nmol/AG)	NA (nmol/AG)	Ad (nmol/AG)
Pnmt-KO Vehicle (NaCl 0.9%)	0.13 ± 0.05	8.41 ± 1.06	Undetectable
Pnmt-KO Ad (0.01 mg/kg)	0.04 ± 0.01	11.55 ± 1.80	Undetectable
Pnmt-KO Glucose (10 mg/kg)	0.13 ± 0.04	0.30 ± 0.03	Undetectable
Pnmt-KO Ad (0.01 mg/kg) + Glucose (10 mg/kg)	0.08 ± 0.04	0.31 ± 0.03	Undetectable

Values are means ± standard error of the means (SEM) of 5–7 mice per group. Pnmt-KO Glucose, adrenaline-deficient mice treated with glucose; Pnmt-KO Ad, adrenaline-deficient mice administered with adrenaline; Pnmt-KO glucose + Ad, adrenaline-deficient mice administered with glucose plus adrenaline; Pnmt-KO vehicle, adrenaline-deficient mice administered with vehicle; DA, dopamine; NA, noradrenaline; Ad, adrenaline

## Discussion

Briefly, the presented results reinforce the view that glucose may have an important role in contextual fear memory even when Ad is absent. In addition, glucose and Ad appear to act in synergy in contextual fear memory strengthening.

After a stressful event, Ad is released into the bloodstream and appears to be essential for fear and traumatic memories strengthening. This has been demonstrated in behavioural models such as inhibitory avoidance [24], contextual fear conditioning [14, 15], and PTSD mouse models [25, 26]. However, due to the biochemical proprieties of this catecholamine, preventing it from crossing the BBB, its actions and effects are thought to be restricted to the periphery [16]. As reviewed in Gold [17], glucose is one of the proposed mediators for Ad effects on memory strengthening. According to this theory, we have shown that Ad released from the medulla zone of the adrenal gland acts in peripheral β_2_-adrenoceptors, activating them [10, 14], and may induce glucose synthesis and release from hepatic tissue [27, 28].

Glucose, the primary energy substrate of the brain, is carried by glucose transport proteins (GLUT-1) across the BBB, where they are found in high density [29]. In the brain, glucose may serve as an energy source for the increased production and release of neurotransmitters into the brain areas highly associated with fear memory, namely the hippocampus or the amygdala, possibly enhancing it. Another set of evidence supporting glucose as a possible Ad mediator in the CNS is that Ad fails to facilitate rat’s performance in a spontaneous alternation task, when the animals were submitted to food-restriction protocols and thus were hypoglycaemic [30]. Besides, adrenergic antagonists are capable of preventing contextual fear memory-strengthening effects induced by Ad [10, 14] but not by glucose [31].

Under the circumstances of our experimental conditions (protocol in Fig. 1A), the chosen dose of glucose induced a moderate hyperglycaemic state in our animals 10 min after administration, as previously described [32]. In mice, the administration of glucose (30 mg/kg) immediately after inhibitory avoidance training strengthens the aversive memory of the aversive situation [32], however, the passive avoidance task does not allow for an accurate investigation of associative memory, contrary to fear conditioning [33].

In the experimental protocol of Fig. 1B, WT mice were administered with glucose (30 mg/kg) only before the fear acquisition session of the fear conditioning protocol, and no differences were observed in fear memory when mice were re-exposed to context. However, when glucose was administered both before the fear acquisition day and re-exposure to the context day (Fig. 1C) the outcome was different: WT mice administered with glucose presented increased contextual fear memory when compared to WT mice administered with the vehicle. This could indicate that glucose in both fear acquisition and context re-exposition may be necessary for fear contextual memory consolidation and reconsolidation. It has been proposed that each phase of the fear conditioning paradigm may be associated with a phase of memory establishment: the training day appears to be strongly related to acquisition (learning), while the context day is more intertwined with retrieval and reconsolidation processes [34, 35]. Because we only observed significant effects when glucose was administered on both days of the paradigm, and not when it was only administered before the acquisition day, we propose that glucose may be crucial for the recall of contextual fear memory.

Considering the Ad levels in the adrenal gland, which is the production site of about 80% of Ad [36], mice administered with glucose only on the acquisition day showed increased levels of this catecholamine when compared with mice administered with the vehicle. However, WT mice administered with glucose on both training and context days showed no differences in adrenal gland Ad levels. These results are in line with previous reports showing that during hyperglycaemia, the production and release of Ad from the adrenal gland is not increased [37]. In our experimental design, glucose-treated mice on fear acquisition and context days were evaluated while still hyperglycaemic, explaining our findings.

A low blood glucose sugar level triggers the release of Ad to induce glucose release to the bloodstream. Therefore, Ad is considered a hyperglycaemic agent [38, 39]. On the other hand, our previous reports showed that blood glucose increased in WT mice after the fear acquisition day of the fear conditioning procedure, which was not observed in Ad-deficient mice [14]. Additionally, exogenous administration of Ad in Ad-deficient mice reverts the observed contextual fear memory impairment [14, 15]. Although several interactions between Ad and glucose exist, none of the previously published works isolate the specific role of glucose. After blood glucose increment, astrocytes increase the glucose uptake for the synthesis of glycogen stores. During learning, the activation of neurotransmitter membrane receptors on those cells triggers glycogenolysis and the production of lactate [40]. Therefore, the role of glucose in memory enhancement may be connected with the astrocytic supply of lactate to neurons as a supplemental energy source [41, 42]. To our knowledge, this is the first study that accurately separates the effect of Ad from that of glucose. Following the contextual fear memory paradigm, Ad-deficient mice administered with glucose in both the acquisition and context days presented a strengthening of contextual fear memory when compared with the respective vehicle group. Therefore, glucose may revert the Ad-deficient mice contextual fear memory impairment, being necessary for consolidation and/or recall of contextual fear memory. Correspondingly, glucose appears to have an important role in contextual fear memory even when Ad is absent.

As previously cited, glucose plays a role as a general fuel for brain cells [40, 41]. Accordingly, glucose administration resulted in the release of acetylcholine in the hippocampus during inhibitory avoidance training and spontaneous alternation [43, 44]. This evidence suggests that some memory neurotransmitter modulators, such as acetylcholine, might operate on astrocyte receptors instead of neuronal receptors [45]. Therefore, our findings might follow this evidence: glucose may trigger the hippocampus’s production of acetylcholine, which could subsequently act on astrocyte receptors to enhance contextual fear memory.

Long-term memory formation and enhancement have been related to the NR4A family of transcription factors encoding genes, that comprise the *Nr4a1*, *Nr4a2*, and *Nr4a3* genes [46]. Furthermore, studies confirmed that emotional memory formation is compromised in *Nr4a2*-deficient mutant mice and hippocampal expression of the three NR4A family members has been shown to increase after contextual fear conditioning [46, 47]. The NR4A family of genes code for transcription factors that can trigger the expression of other genes, including the brain-derived neurotrophic factor (*Bdnf*), which has been linked to long-term memory formation [46, 48]. BDNF (peptide encoded by the *Bdnf* gene) is needed for the formation of brain circuits, the development and preservation of neuronal morphology, brain architecture, as well as synaptic and neuronal network plasticity [49–51]. Concerning the formation of fear memories, it has also been shown that fear conditioning causes an increase in *Bdnf* gene expression in the amygdala and is also widely expressed in the hippocampus, implying that this neurotrophin is involved in contextual fear memory and traumatic memories [49, 52].

Our group has previously demonstrated that Ad-deficient mice presenting less recall of a fearful event also presented lower levels of hippocampal *Nr4a* mRNA expression on the fear acquisition day of the contextual fear paradigm. Furthermore, when Ad was administered to the Ad-deficient mice, it was observed an increase in *Nr4a2* mRNA expression [10]. In this work, we assessed the hippocampal mRNA expression of *Nr4a1*, *Nr4a2*, *Nr4a3*, and *Bdnf* after a fear conditioning procedure in Ad-deficient mice administered with glucose (30 mg/kg). *Nr4a3* and *Bdnf* mRNA expression in the hippocampus of Ad-deficient mice treated with glucose (30 mg/kg) was significantly increased in comparison with those administered with the respective vehicle. These results are in line with a previously published article in which hippocampal mRNA expression of *Bdnf* as well as contextual fear memory were increased following insulin administration in Ad-deficient mice [53]. We propose that this happens due to an increase in glucose uptake in the CNS. In addition, our group has shown a possible involvement of the *Nr4a* gene family in the persistence of PTSD symptoms and signs [25], verifying a significant downregulation of the *Nr4a1* gene expression in the hippocampus of WT mice administrated with sotalol, a peripheral β-adrenoceptor antagonist, when compared to vehicle-treated mice [54].

In another set of experiments aiming to evaluate a possible synergy between Ad and glucose, Ad-deficient mice were submitted to the same fear conditioning protocol described above and administered with both sub-effective doses of Ad (0.01 mg/kg) and/or glucose (10 mg/kg) (Fig. 1D). The separate administration of these sub-effective doses was insufficient to strengthen contextual fear memory. However, the simultaneous administration of these sub-effective doses of Ad and glucose proved to strengthen contextual fear memory in Ad-deficient mice. These results lead us to suggest that Ad and glucose might act synergically to improve memory acquisition and consolidation. Altogether, this could indicate a synergy between Ad and glucose when animals are submitted to aversive tasks (such as fear conditioning procedure), contrary to non-aversive tasks which will not induce the release of Ad from the adrenal medulla.

In conclusion, our results reinforce the hypothesis that glucose may be important for the recall of contextual fear memories and a crucial part of the peripheral to the central pathway for the retrieval and reconsolidation of fear memories independently of Ad. These effects possibly occur through the upregulation of *Nr4a3* and *Bdnf* mRNA expression in the hippocampus which may be directly related to central molecular mechanism changes. In addition, Ad and glucose appear to act in synergy to enhance contextual fear memory establishment and persistence.

## Data Availability

All data generated or analyzed in this study are available in the published article.
